# Descriptive epidemiological study of South African colorectal cancer patients at a Johannesburg Hospital Academic institution

**DOI:** 10.1002/jgh3.12248

**Published:** 2019-09-30

**Authors:** Michelle McCabe, Yvonne Perner, Rindidzani Magobo, Sheefa Mirza, Clement Penny

**Affiliations:** ^1^ Department of Anatomical Pathology, School of Pathology, Faculty of Health Sciences University of the Witwatersrand Johannesburg South Africa; ^2^ Department of Internal Medicine, Faculty of Health Sciences University of the Witwatersrand Johannesburg South Africa

**Keywords:** colorectal cancer, descriptive epidemiology, race, South Africa

## Abstract

**Background and Aim:**

Epidemiological studies of colorectal cancer (CRC) in South Africa (SA) have been poorly characterized. Black and white SA population groups have demonstrated distinct CRC clinical presentations, suggesting that black SA patients follow a different carcinogenic pathway than their white counterparts. Thus, the aim of this study was to identify unique demographic and histopathological features associated with black SA patients to facilitate earlier diagnosis and to improve disease management.

**Methods:**

This preliminary descriptive epidemiological study included 665 retrospective CRC cases diagnosed between the period 2011 and 2015 at the Charlotte Maxeke Johannesburg Academic Hospital. Demographic and histopathological features in black *versus* other race groups (ORG) were compared, and Student's *t*‐test, Chi‐square, and Fischer's exact tests were used for statistical analysis.

**Results:**

Statistical analysis demonstrated that patients with left‐sided tumors of invasive adenocarcinoma were predominantly black and male. These patients were considerably younger when compared to ORG (median 56 *vs* 62 years, respectively), *P* < 0.0001. However, no significant propensity for other histological features was illustrated. Polyps were mostly tubular adenomas (51%) and tubulovillous adenomas (TVAs) (44%). TVAs were mostly high‐grade lesions (*P* < 0.0001) and associated with left‐sided CRC (*P* = 0.0325).

**Conclusion:**

These findings verify that black SA CRC patients have an earlier disease onset in comparison to ORG; however, no increased tendency for tumor site, precursor lesion, stage of disease, or gender was evident. Thus, a deeper molecular characterization of CRC is required to understand the underlying causes associated with earlier disease onset in black SA CRC patients.

## Graphical abstract

This study demonstrated that black South Africa (SA) colorectal cancer (CRC) patients present at a significantly younger age in comparison to other SA race groups; however, no increased tendency for tumor site, precursor lesion, stage of disease, or gender was shown with regard to race. CRC in black SA patients also appears to be distinct from African American patients, illustrating a different mechanism of disease and characterization at the molecular level required to understand the underlying pathology.

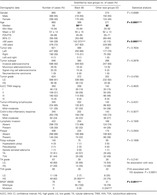

## Introduction

Colorectal cancer (CRC) is the third most common cancer worldwide and the fourth most common cause of cancer‐related mortalities.^1^ Within South Africa (SA), it is the fourth most commonly diagnosed cancer, being the second most common among males and the fourth most common among females and the sixth leading cause of cancer mortalities in SA.^1^, ^2^ Male and female ratios have remained constant (53–55% *vs* 45–47%, respectively).^2^, ^3^, ^4^, ^5^, ^6^ Male patients have a 1.27 estimated lifetime risk of developing CRC, and females have a lower risk of 0.75.^6^ CRC incidence rates within SA have progressively increased over the years, with the annual crude incidence in SA CRC male and female patients in 2014 reported as 7.34 and 5.86 per 100 000, respectively.^6^ CRC occurs mostly within the white (Caucasian) population group in SA patients (52–54%), followed by black (African) (26–28%), colored (mixed ancestry) (14–15%), and Asian (Indian) patients (4–7%).^2^, ^3^, ^4^, ^5^, ^6^ The pathogenesis of CRC is complex and diverse and is influenced by multiple factors, including diet, lifestyle, and genetic predispositions. It is reported that the white patient population group in SA appears to follow the classic Western trend, which presents at a later age, having an association with diet and lifestyle factors, although the molecular pathology has not been extensively investigated.^7^, ^8^ Comparatively, the black SA population has a higher frequency of young (<50 years) CRC patients, possibly because of diet and lifestyle changes either due to urbanization or a familial contribution.^7^, ^8^, ^9^ A study by Prodehl *et al*. showed that 49.0% of SA CRC patients reported a family history of cancer, with CRC the most frequently diagnosed, where maternal (35%) and fraternal (33%) members were the most affected, followed by paternal members (25%).^10^

High prevalence rates for young CRC patients, <40 years of age, have been reported globally, with the lowest rates found in the United States, Europe, and New Zealand (1–6%) and the highest in Asian and Middle Eastern countries (10–39%).^11^, ^12^, ^13^ The current SA National Cancer Registry (NCR) statistics, spanning a 5‐year period (2010–2014), show that young CRC patients, under 50 years of age, contribute an average of 18% of all CRC cases, of which approximately 7% were younger than 40 years of age.^2^, ^3^, ^4^, ^5^, ^6^ The NCR 2014 report also showed an exponential growth in CRC incidence in male patients from 2010 to 2014. A correlation between race and age was also demonstrated as the majority of young patients younger than 50 years old were black (32%) *versus* white patients (11%) (*P* < 0.0001) (NCR 2013–2014).^5^, ^6^ The burden of disease in SA has also been a challenge to assess due to underreporting of CRC cases as registries are mainly limited to small urban areas.^14^

Thus, this descriptive epidemiological study will serve as a preliminary study to obtain a greater understanding of the development of CRC, particularly in black SA CRC patients. This will include screening demographic and histopathological data in the hope of identifying key features associated with the disease to assist with earlier detection and improvement of prognosis, particularly within this population group.

## Methods

A retrospective laboratory information system (LIS) search was conducted on all patients who had biopsy samples or colorectal resections reported by the Charlotte Maxeke Johannesburg Academic Hospital (CMJAH) branch of the National Health Laboratory Service (NHLS)/Anatomical Pathology Division, Faculty of Health Sciences, University of the Witwatersrand. Histological reports of all patients diagnosed with colorectal adenocarcinoma between 2011 and 2015 were reviewed, and data were described with respect to age, race, gender, tumor site ([distal to the splenic flexure [left‐sided] and proximal to the splenic flexure [right‐sided]), histological subtype and grade, presence and grade of precursor lesion, invasive tumor including the presence of tumor infiltrating lymphocytes (TIL), Crohn's‐like inflammatory reaction (CIR), and the tumor stage (TNM). The Ethics Committee of the University of the Witwatersrand (clearance number M120994) approved this study.

### *Statistical analysis*

Statistical associations between race groups were investigated using Student's *t*‐test, Fisher's exact test, and Chi Square test analysis. Non‐black race patients were included in a single group, termed the “Other Race” group (White, Colored/mixed ancestry, Indian) due to small case numbers in the colored and Indian groups in comparison to the black patient group. Race was compared with age, tumor site, and morphological features as stated above. Statistical data analysis was completed using Stata Intercooled 7.0 (Stata, College Station, TX, USA) and Graphpad Prism version 7 (GraphPad Software, La Jolla, CA, USA). The results were considered statistically significant when *P* < 0.05* and highly significant when *P* < 0.001***.

## Results

A total of 665 CRC patients were diagnosed at CMJAH between 2011 and 2015, with the majority of patients being black (391/665; 59%) (Table 1). Overall, the patients were predominantly male (366/665; 55%), and no variation of gender was seen among race groups. The median age of CRC was 59 years (interquartile range: 49–68). A significant association was seen between patient age and race (black *vs* other race group [ORG]) (median age: 56 *vs* 62 respectively), (*P* < 0.0001). Most tumors occurred in the left colon (427/621; 69%), had an invasive adenocarcinoma subtype (596/646; 92%), were low‐grade (LG) (568/624; 91%), and presented as TNM stage III (180/350; 52%). The majority of CRC cases did not contain TILs (224/345; 65%) or a CIR (263/345; 76%), nor did they show lymphatic invasion (296/422; 70%). A minority of polyps was found (140/408; 34%), with tubular adenomas (TAs) being reported as the predominant subtype (72/140; 51%), followed by tubulovillous adenomas (TVAs) (61/140; 44%). TAs mostly displayed LG dysplasia (40/67; 60%); however, TVAs were mostly associated with high‐grade (HG) dysplasia (49/60; 82%) (*P* < 0.0001). All the aforementioned pathological characteristics lacked any correlation with race. BRAF V600E mutations, however, exclusively occurred in ORG patients (5/21; 24%) (*P* = 0.0011).

**Table 1:** Descriptive histopathological analysis of colorectal cancer cases diagnosed at Charlotte Maxeke Johannesburg Academic Hospital between 2011 and 2015: Black *versus* other race groups

		Stratified by race groups no. of cases (%)		
Demographic data	Number of cases (%)	Black (B)	Other race groups (O)	Statistical analysis:
Gender	665	391	274	*P* = 0.9369
Male	366 (55)	216 (55)	150 (55)	
Female	299 (45)	175 (45)	124 (45)	
Age	660	389	271	***P* < 0.0001** ***
Median	59	56***	62	
Min–Max	15–94	15–94	25–92	
Mean ± SD	57 ± 14	55 ± 15	62 ± 13	
P25‐P75	49–68	45–65	55–71	
95% CI	[56–59]	[53–56]	[60–64]	
≤50 years	184 (28)	142 (37)***	42 (15)	***P* < 0.0001** ***
>50 years	476 (72)	247 (63)	229 (85)	
Tumor site	621	368	253	*P* = 0.7634
Left	427 (69)	250 (68)	177 (70)	
Right	190 (30)	115 (31)	75 (30)	
Left and right	4 (1)	3 (1)	1 (0)	
Tumor subtype	646	380	266	*P* = 0.2878
Invasive adenocarcinoma	596 (92)	349 (92)	247 (93)	
Mucinous adenocarcinoma	29 (5)	16 (4)	13 (5)	
Signet ring cell adenocarcinoma	20 (3)	15 (4)	5 (2)	
Neuroendocrine carcinoma	1 (0)	0 (0)	1 (0)	
Tumor grade	624	372	252	*P* = 0.4793
LG	568 (91)	336 (90)	232 (92)	
HG	56 (9)	36 (10)	20 (8)	
AJCC TNM staging	350	208	142	*P* = 0.4625
I	46 (13)	26 (13)	20 (15)	
II	109 (31)	59 (28)	50 (35)	
III	180 (52)	114 (55)	66 (46)	
IV	15 (4)	9 (4)	6 (4)	
Tumor‐infiltrating lymphocytes	345	202	143	*P* = 0.4231
None	224 (65)	135 (67)	89 (62)	
Mild–moderate	121 (35)	67 (33)	54 (38)	
Crohn's‐like inflamatory response	345	202	143	*P* = 0.3077
None	263 (76)	158 (78)	105 (73)	
Mild–moderate	82 (24)	44 (22)	38 (27)	
Lymphatic invasion	422	254	168	*P* = 0.1935
Absent	296 (70)	172 (68)	124 (74)	
Present	126 (30)	82 (32)	44 (26)	
Polyps	408	234	174	*P* = 0.2934
Absent	268 (66)	160 (68)	108 (62)	
Present	140 (34)	74 (32)	66 (38)	
Polyp subtype	140	75	65	*P* = 0.1856
Hyperplastic polyp	4 (3)	1 (1)	3 (5)	
Pseudopolyp	2 (1)	2 (3)	0 (0)	
Sessile serrated adenoma	1 (1)	0 (0)	1 (1)	
TA	72 (51)	43 (57)	29 (45)	
TVA	61 (44)	29 (39)	32 (49)	
TA grade	67	39	28	*P* = 0.2161
LG	40 (60)	25 (65)	15 (54)	
HG	27 (40)	14 (35)	13 (46)	
TVA grade	60	28	32	
LG	11 (18)	2 (7)	8 (25)	
HG	49 (82)	25 (93)***	24 (75)	
BRAF V600E	76	55	21	***P* = 0.0011** ***
Wild‐type	71	55 (100)	16 (76)	
Mutation	5	0 (0)	5 (24)***	

*Statistically significant.

***Statistically highly significant.

AJCC, American Joint Committee on Cancer; CI, confidence interval; HG, high grade; LG, low grade; TA, tubular adenoma; TNM, tumor node metastases; TVA, tubulovillous adenoma.

Correlation between age groups, young *versus* old (≤50 *vs* >50 years) and CRC subtype, tumor grade, TIL, and lymphatic invasion was identified (Table 2). Signet ring cell carcinoma occurred at a higher frequency in the young patient group (16/183; 9%) *versus* the older patient group (4/463; 1%) (*P* = 0.0030), while a mild to moderate TIL response was more commonly found in the older patient group (95/247; 38%) than in the younger group (26/98; 27%) (*P* = 0.0450). Lymphatic invasion was associated more with younger patients (45/120; 37%) than with the older group (81/302; 27%) (*P* = 0.0341), and BRAF V600E mutations were exclusively found in older patients (5/36; 24%) *versus* younger patients (0/40; 0%) (*P* = 0.0204). No significant association was found between gender and histopathological data (Table 3).

**Table 2:** Descriptive histopathological analysis of colorectal cancer cases diagnosed at Charlotte Maxeke Johannesburg Academic Hospital between 2011 and 2015: Younger (≤50 years) *versus* older (>50 years) patients

		Stratified by age groups no. of cases (%)		
Demographic data	Number of cases (%)	≤50 years	>50 years	Statistical analysis:
Gender	665	189	476	*P* = 0.1005
Male	366 (55)	114 (60)	252 (53)	
Female	299 (45)	75 (40)	224 (47)	
Age	660	389	271	***P* < 0.0001** ***
Median	59	41	64	
Min–Max	15–94	15–50	51–94	
Mean ± SD	57 ± 14	40 ± 8	65 ± 9	
P25‐P75	49–68	34–46	57–71	
95% CI	[56–59]	[39–41]	[64–66]	
Tumor site	621	179	442	*P* = 0.2999
Left	426 (68)	119 (66)	307 (69)	
Right	191 (31)	60 (34)	131 (30)	
Left and right	4 (1)	0 (0)	4 (1)	
Tumor subtype	646	183	463	***P* < 0.0001** ***
Invasive adenocarcinoma	596 (92)	159 (87)	437 (94)	
Mucinous adenocarcinoma	29 (5)	8 (4)	21 (5)	
Signet ring cell adenocarcinoma	20 (3)	16 (9)***	4 (1)	
Neuroendocrine carcinoma	1 (0)	0 (0)	1 (0)	
Tumor grade	624	178	446	*P* = 0.0030**
LG	568 (91)	152 (85)	416 (93)	
HG	56 (9)	26 (15)**	30 (7)	
AJCC TNM staging	350	100	250	*P* = 0.2282
I	46 (13)	9 (9)	37 (15)	
II	109 (31)	27 (27)	82 (33)	
III	180 (52)	59 (59)	121 (48)	
IV	15 (4)	5 (5)	10 (4)	
Tumor‐infiltrating lymphocytes	345	98	247	***P* = 0.0450** *
None	224 (65)	72 (73)	152 (62)	
Mild–moderate	121 (35)	26 (27)	95 (38)*	
Crohn's‐like inflamatory response	345	98	247	*P* = 0.1613
None	263 (76)	80 (82)	183 (74)	
Mild–moderate	82 (24)	18 (18)	64 (26)	
Lymphatic invasion	422	120	302	***P* = 0.0341** *
Absent	296 (70)	75 (63)	221 (73)	
Present	126 (30)	45 (37)*	81 (27)	
Polyps	408	111	297	*P* = 0.1610
Absent	268 (66)	79 (71)	189 (64)	
Present	140 (34)	32 (29)	108 (36)	
Polyp subtype	140	32	108	*P* = 0.6291
Hyperplastic polyp	4 (3)	0 (0)	4 (4)	
Pseudopolyp	2 (1)	1 (3)	1 (1)	
Sessile serrated adenoma	1 (1)	0 (0)	1 (1)	
TA	72 (51)	18 (56)	54 (50)	
TVA	61 (44)	13 (41)	48 (44)	
Polyp grade				*P* = 0.6828
TA	67	18	49	
LG	40 (60)	10 (56)	30 (60)	
HG	27 (40)	8 (44)	19 (40)	
TVA	60	12	48	
LG	11 (18)	3 (25)	8 (17)	
HG	49 (82)	9 (75)	40 (83)	
BRAF V600E	76	40	36	***P* = 0.0204** *
Wildtype	71	40 (100)	31 (76)	
Mutation	5	0 (0)	5 (24)*	

*Statistically significant.

***Statistically highly significant.

AJCC, American Joint Committee on Cancer; CI, confidence interval; HG, high grade; LG, low grade; TA, tubular adenoma; TNM, tumor node metastases; TVA, tubulovillous adenoma.

**Table 3:** Descriptive histopathological analysis of colorectal cancer cases diagnosed at Charlotte Maxeke Johannesburg Academic Hospital between 2011 and 2015: Male *versus* female

		Stratified by gender no. of cases (%)		
Demographic data	Number of cases (%)	Male	Female	Statistical analysis:
Gender	665	366 (55%)	299 (45%)	
Age	660	364	296	*P* = 0.2560
Median	59	58	60	
Min–Max	15–94	15–94	20–92	
Mean ± SD	57 ± 14	56 ± 14	59 ± 14	
P25‐P75	49–68	47–67	51–69	
95% CI	[56–59]	[55–58]	[58–61]	
Tumor site	621	344	277	*P* = 0.9653
Left	426 (68)	237 (69)	189 (68)	
Right	191 (31)	105 (31)	86 (31)	
Left and right	4 (1)	2 (0)	2 (1)	
Tumor subtype	646	183	463	*P* = 01663
Invasive adenocarcinoma	596 (92)	437 (87)	267 (94)	
Mucinous adenocarcinoma	29 (5)	21 (4)	14 (5)	
Signet ring cell adenocarcinoma	20 (3)	4 (9)	9 (1)	
Neuroendocrine carcinoma	1 (0)	1 (0)	0 (0)	
Tumor grade	624	344	280	*P* = 01611
LG	568 (91)	308 (85)	260 (93)	
HG	56 (9)	36 (15)	20 (7)	
AJCC TNM staging	350	100	250	*P* = 0.2282
I	46 (13)	9 (9)	37 (15)	
II	109 (31)	27 (27)	82 (33)	
III	180 (52)	59 (59)	121 (48)	
IV	15 (4)	5 (5)	10 (4)	
Tumor‐infiltrating lymphocytes	345	192	153	*P* = 0.1119
None	224 (65)	132 (69)	92 (60)	
Mild–moderate	121 (35)	60 (31)	61 (40)	
Crohn's‐like inflamatory response	345	192	153	*P* = 0.0569
None	263 (76)	154 (80)	109 (71)	
Mild–moderate	82 (24)	38 (20)	44 (29)	
Lymphatic invasion	422	234	188	*P* = 0.6698
Absent	296 (70)	162 (69)	134 (71)	
Present	126 (30)	72 (31)	54 (29)	
Polyps	408	236	174	*P* = 0.0917
Absent	268 (66)	147 (62)	123 (71)	
Present	140 (34)	89 (38)	51 (29)	
Polyp subtype	140	89	51	*P* = 0.4160
Hyperplastic polyp	4 (3)	3 (4)	1 (2)	
Pseudopolyp	2 (1)	2 (2)	0 (0)	
Sessile serrated adenoma	1 (1)	0 (0)	1 (2)	
TA	72 (51)	43 (48)	29 (57)	
TVA	61 (44)	41 (46)	20 (39)	
Polyp grade				*P* = 0.4732
TA	67	36	28	
LG	40 (60)	21 (58)	17 (54)	
HG	27 (40)	15 (42)	11 (46)	
TVA	60	41	19	
LG	11 (18)	6 (15)	5 (28)	
HG	49 (82)	35 (85)	14 (72)	
BRAF V600E	76	44	32	*P* = 0.6444
Wildtype	71	42 (95)	29 (91)	
Mutation	5	2 (5)	3 (9)	

*Statistically significant.

***Statistically highly significant.

AJCC, American Joint Committee on Cancer; CI, confidence interval; HG, high grade; LG, low grade; TA, tubular adenoma; TNM, tumor node metastases; TVA, tubulovillous adenoma.

The majority of polyps were found in the left colon (81/131; 62%) and in male patients (89/140; 64%) (Tables 3 and 4). A particular association between the side of colon and grade of TVAs was noted here (*P* = 0.0325) as left‐sided CRCs were mostly associated with HG TVAs (31/34; 91%). BRAF V600E mutations occurred exclusively in the right colon (5/34; 15%), and none were associated with left CRC tumors (0/42; 0%) (*P* = 0.0151).

**Table 4:** Descriptive histopathological analysis of CRC cases diagnosed at Charlotte Maxeke Johannesburg Academic Hospital between 2011–2015: Left *versus* right‐sided colon cancer

		Stratified by tumor site no. of cases (%)		
Demographic data	Number of cases (%)	Left‐sided	Right‐sided	Statistical analysis:
Tumor site	617	426 (69%)	191 (31%)	
Tumor subtype	607	419	188	***P* < 0.0001** ***
Invasive adenocarcinoma	596 (92)	398 (95)	162 (86)	
Mucinous adenocarcinoma	29 (5)	9 (2)	18 (10)***	
Signet ring cell adenocarcinoma	20 (3)	12 (3)	7 (4)	
Neuroendocrine carcinoma	1 (0)	0 (0)	1 (0)	
Tumor grade	587	399	188	*P* = 02845
LG	533 (91)	366 (92)	167 (89)	
HG	54 (9)	33 (8)	21 (11)	
AJCC TNM staging	350	192	153	*P* = 0.6086
I	46 (13)	28 (15)	17 (11)	
II	109 (31)	60 (31)	46 (30)	
III	180 (52)	96 (50)	80 (52)	
IV	15 (4)	8 (4)	10 (7)	
Tumor‐infiltrating lymphocytes	338	193	145	***P* = 0.0210** *
None	220 (65)	136 (70)	84 (58)	
Mild–moderate	118 (35)	57 (30)	61 (42)*	
Crohn's like inflamatory response	338	193	145	*P* = 1.0000
None	258 (76)	147 (76)	111 (77)	
Mild–moderate	80 (24)	46 (24)	34 (23)	
Lymphatic invasion	417	254	163	***P* = 0.0009** ***
Absent	293 (70)	194 (76)	99 (61)	
Present	123 (30)	60 (24)	64 (39)***	
Polyps	392	231	161	*P* = 0.4468
Absent	261 (67)	150 (65)	111 (69)	
Present	131 (33)	81 (35)	50 (31)	
Polyp subtype	131	81	50	*P* = 0.1484
Hyperplastic polyp	3 (2)	3 (4)	0 (0)	
Pseudopolyp	2 (1)	0 (0)	2 (4)	
Sessile serrated adenoma	1 (1)	0 (0)	1 (2)	
TA	68 (52)	43 (53)	25 (50)	
TVA	57 (44)	35 (43)	22 (44)	
Polyp grade	119			*P* = 0.0615
TA	64	40	24	
LG	38 (59)	21 (52)	17 (71)	
HG	26 (41)	19 (48)	7 (29)	
TVA	55	34	21	
LG	10 (18)	3 (9)	7 (33)	
HG	45 (82)	31 (91)*	14 (67)	
BRAF V600E	76	42	34	***P* = 0.0154** *
Wildtype	71	42 (100)	29 (85)	
Mutation	5	0 (0)	5 (15)*	

*Statistically significant.

***Statistically highly significant.

AJCC, American Joint Committee on Cancer; CI, confidence interval; CRC, colorectal cancer; HG, high grade; LG, low grade; TA, tubular adenoma; TNM, tumor node metastases; TVA, tubulovillous adenoma.

## Discussion

The focus of this study was to ascertain common features, particularly demographic and histopathological characteristics, associated with black patients to assist in the earlier detection and better management of the disease. Although this study demonstrated no significant differences between male and female patients, black *versus* ORG and younger *versus* older patient groups illustrated significant differences in a number of parameters.

A total of 665 CRC patients were diagnosed at CMJAH over a 5‐year period, from 2011 to 2015. Patients were predominantly male and black, with a considerable proportion being young. The median age of black patients compared to ORG patients (56 *vs* 62 years, respectively) was significantly younger. The data confirmed local and international reports regarding male *versus* female ratios (55 *vs* 45%, respectively),^1^, ^5^ with black patients showing an earlier age of onset in comparison to the white group.^7^, ^15^ In comparison, in African Americans (AA), the median age of onset in males and females were reported to be 66 and 70 years, respectively, compared to 72 and 77 in white men and women, respectively.^16^ In this SA cohort, the median age for male and female black patients was 55 and 56 years, respectively, *versus* 62 and 65 years for males and females of ORG, respectively. The median age for white male and female patients (separated from Indian and colored or mixed race) remained 62 and 65 years, respectively. These data showed that SA black and white patients are at least 10 years younger in comparison to U.S. AA and U.S. white patients.

The frequency of young black patients younger than 50 years of age in this cohort (37%) was similar to that of young black SA CRC patients in the NCR 2014 report (32%). The majority of tumors occurred in the left colon, had an invasive adenocarcinoma subtype, LG tumors, and presented at an advanced TNM stage (III and IV) These findings were in concordance with international published data that reported an approximate 70% occurrence of left‐sided CRC, with about 90% being of an invasive adenocarcinoma subtype, that was moderately to well differentiated, with approximately 50–60% of CRC cases presenting at a more advanced stage.^17^, ^18^ Male and female patients had equal frequencies for tumor site (69% left‐sided *vs* 31% right‐sided). This finding is contrary to other studies showing right‐sided colon cancer to be increased in female patients and left‐sided CRC to be increased in males.^19^

In the present analysis, with regard to polyps, conventional adenomas (TA, TVA, and villous adenomas [VAs]) are mostly found in the left colon,^20^ with TAs described as the predominant subtype (80%), TVAs accounting for 10–20%, and VAs less than 5%.^21^, ^22^, ^23^ In this study, polyps were found in 34% (140/408) of cases, with only 1% being sessile serrated adenoma (SSA), 4% hyperplastic polyps (HPs), 51% TAs, and 44% TVAs. TAs were mostly associated with LG dysplasia (60%), and TVAs that also occurred at a considerably high frequency than that stated in literature were mostly associated with HG dysplasia (83%) (*P* < 0.0001). The size of the adenomatous polyp (AP) was not recorded in the majority of reports, only the quantity of APs and grade of dysplasia. This study showed no association of APs with race, gender, or patient age; however, HG TVAs were more commonly found in the left colon (*P* = 0.0325). The transition from LG APs to more advanced APs or CRC takes approximately 3 years, suggesting that frequent surveillance colonoscopies for the earlier detection of CRC would benefit patients. Guidelines indicate repeat surveillance every 5–10 years for 1–2 small TAs (<1 cm) with LG dysplasia, and where 3–10 large APs are found (≥1 cm) or in those with HG dysplasia or with villous features, colonoscopy should be performed every 3 years.^20^, ^23^

The literature indicates that AA patients are more likely to have right‐sided tumors than left.^15^, ^24^, ^25^ This SA study, however, showed no relationship between race and tumor site. BRAF V600E mutational analysis was carried out in 76 patients, and an association was only found in older white patients with right‐sided colon cancers, possibly indicating development via the microsatellite instability CRC pathway.

CRC research in AA patients has demonstrated higher incidence and mortality rates, earlier age of diagnosis, more advanced stage of the disease, and a greater proportion of right‐sided colon cancers in comparison to other ethnic groups. This has led American professional societies to change guidelines, recommending colonoscopy screening by 45 instead of 50 years of age, which has led to a great reduction in CRC incidence in this population group.^26^ Although statistically significant differences concerning stage and tumor site were not seen when comparing black *versus* ORG patients, later stage of disease and earlier age of onset in black patients remained significant factors, and a policy recommending earlier screening in this population group should be considered to reduce incidence rates.

In conclusion, this study shows that black SA CRC patients most likely follow a different carcinogenic pathway in comparison to other SA ethnic groups due to earlier age of onset. Black SA patients also seem to present at least a decade earlier when comparing Africans found in the Western world, with more left‐sided CRC occurring in SA patients compared to a right‐sided CRC association seen in AAs. Moreover, a lack of BRAF V600E mutation in the black SA CRC population in this study suggests that there is a different carcinogenic pathway in black SA CRC patients compared to other ethnic groups, which still needs to be evaluated in a larger cohort of patients. TVAs found at a frequent rate across different ethnic groups in SA patients could possibly suggest a dominant adenocarcinoma sequence in the development of the disease. The initiation of earlier screening and more frequent surveillance policies in black SA patients could possibly result in better management of the disease. Further epidemiological research linked to molecular characterization in SA population groups is still needed to assist in determining the underlying cause and designing more personalized treatment strategies for better management of the disease.
